# Viral Delivery of Antioxidant Genes as a Therapeutic Strategy in Experimental Models of Amyotrophic Lateral Sclerosis

**DOI:** 10.1038/mt.2013.115

**Published:** 2013-06-04

**Authors:** Aikaterini Nanou, Adrian Higginbottom, Chiara F Valori, Matthew Wyles, Ke Ning, Pamela Shaw, Mimoun Azzouz

**Affiliations:** 1 Department of Neuroscience, Sheffield Institute for Translational Neuroscience (SITraN), University of Sheffield, Sheffield, UK; 2 Faculty of Medicine, King Abdulaziz University, Jeddah, Saudi Arabia; 3 Current Addresses: Biomedical Research Foundation of the Academy of Athens (BRFAA), Athens, Greece; 4 German Center for Neurodegenerative Diseases (DZNE), Tübingen, Germany

## Abstract

Amyotrophic lateral sclerosis (ALS) is a progressive neurodegenerative disorder with no effective treatment to date. Despite its multi-factorial aetiology, oxidative stress is hypothesized to be one of the key pathogenic mechanisms. It is thus proposed that manipulation of the expression of antioxidant genes that are downregulated in the presence of mutant SOD1 may serve as a therapeutic strategy for motor neuronal protection. Lentiviral vectors expressing either *PRDX3* or *NRF2* genes were tested in the motor neuronal-like NSC34 cell line, and in the ALS tissue culture model, NSC34 cells expressing the human SOD1^G93A^ mutation. The NSC34 SOD1^G93A^ cells overexpressing either *PRDX3* or *NRF2* showed a significant decrease in endogenous oxidation stress levels by 40 and 50% respectively compared with controls, whereas cell survival was increased by 30% in both cases. The neuroprotective potential of those two genes was further investigated *in vivo* in the SOD1^G93A^ ALS mouse model, by administering intramuscular injections of adenoassociated virus serotype 6 (AAV6) expressing either of the target genes at a presymptomatic stage. Despite the absence of a significant effect in survival, disease onset or progression, which can be explained by the inefficient viral delivery, the promising *in vitro* data suggest that a more widespread CNS delivery is needed.

## Introduction

Amyotrophic lateral sclerosis (ALS) or motor neuron disease is a progressive neurodegenerative disorder that is characterized by the loss of upper and lower motor neurons of the spinal cord, brain stem and motor cortex. Approximately 5–10% of ALS cases are inherited in an autosomal dominant fashion, of which 15–20% of cases are caused by mutations in *SOD1* (Cu/Zn superoxide dismutase 1). Although ALS pathogenic mutations have been found in 19 genes and mutations in the newly identified *C9ORF72* gene represent the most common cause of both familial and sporadic ALS,^1,2^ the *SOD1* gene has been more widely studied and used to model ALS. In the presence of mutant SOD1, multiple interacting factors contribute to motor neuron injury including protein misfolding and aggregation, defective axonal transport, excitotoxicity, mitochondrial dysfunction, dysregulated transcription and RNA processing, endoplasmic reticulum stress, apoptosis, oxidative stress, and even toxicity caused by non-neuronal cells.^3^

Of these mechanisms, oxidative stress is considered to play a key role. Biochemical indices of free radical damage have been observed in cerebrospinal fluid and human post-mortem CNS tissue of both familial and sporadic ALS cases.^4,5^ In addition, oxidative damage has been observed in proteins,^5^ lipids,^6^ DNA,^7^ and also RNA species^8^ in post-mortem tissues from both familial and sporadic ALS cases. Furthermore, mutant SOD1 in microglia and astrocytes enhanced nitrogen oxides oxidation resulting in reactive oxygen species production, and treatment with a nitrogen oxides inhibitor not only attenuated glial cell toxicity, but also increased significantly the life span of ALS mice.^9^ Thus, a therapeutic strategy delivering effective antioxidants could provide significant protection of motor neurons in ALS.

Through both a genomic and a proteomic approach several genes have been identified, the expression of which is downregulated in ALS models and the human disease. Two of them (*NRF2* and *PRDX3*) were chosen to be manipulated in their expression as a therapeutic strategy for the neuroprotection of motor neurons and the two genes are antioxidant response element (*ARE*) genes.^10,11,12^ Peroxiredoxin 3 (*PRDX3*) is a mitochondrial thioredoxin–dependent hydroperoxidase, which catalyzes the reduction of peroxides in the presence of thioredoxin, and it is upregulated in response to oxidative stress.^13^ In particular, it directly interacts with the mitochondrial thioredoxin 2, and this system functions in parallel to the glutathione system to protect mitochondria from oxidative stress.^14^ Reduced PRDX3 expression may contribute to the mitochondrial morphological abnormalities seen in cellular and transgenic mouse models of ALS.^15^ Evidence of the neuroprotective ability of PRDX3 came from a study investigating Ebselen, a small molecule antioxidant drug that acts as a peroxiredoxin mimic and offers neuroprotection by inducing transcription of *ARE* genes.^12^

Nuclear factor erythroid 2–related factor 2 (*NRF2*) is a basic leucine zipper transcription factor and is the principal component leading to ARE-mediated gene expression, a motif that is found in the promoter regions of detoxifying genes and antioxidant enzymes.^16^ NRF2 is sequestered in the cytoplasm by the actin-bound protein KEAP1.^17,18^ NRF2 regulates many phase 2 detoxifying enzymes, and at least 14 genes controlled by NRF2 have been shown to be downregulated in the presence of mutant SOD1 including antioxidant genes, and also genes involved in DNA repair, immune response and signaling.^19,20^ The mRNA and protein levels were decreased in the presence of mutant SOD1 transfected cells, and similar findings were shown in primary motor cortex and spinal cord post-mortem tissue samples from patients with ALS with a reduction of NRF2 at both protein and mRNA levels, and a concomitant increase in *KEAP1* mRNA levels.^21^ Importantly, specific NRF2 overexpression in astrocytes of ALS mice resulted in decreased glial reactivity, delayed disease onset and increased mouse survival.^22^

Thus, these two antioxidant genes (*PRDX3* and *NRF2*) were delivered through a lentiviral system in tissue culture models of ALS to assess their effect on cell survival and cellular oxidative stress levels. Both genes were then taken to the mouse model of the disease, as their viral delivery in cells showed decreased levels of oxidative stress by 50% and increased cell survival by 30%, with ultimate goal the development of an efficient therapeutic strategy.

## Results

### LV-mediated neuroprotection in neuronal cells *in vitro*

**Figure 1:**
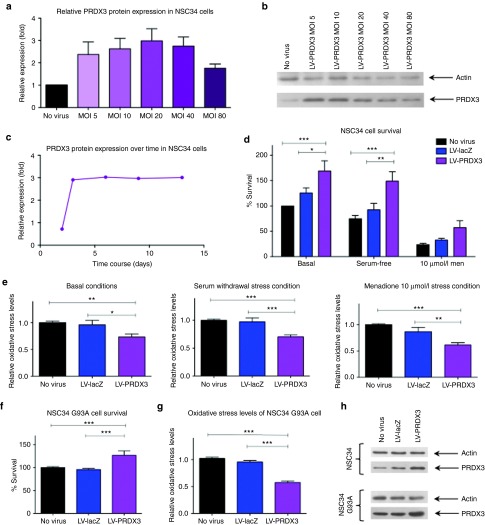
**LV-PRDX3–mediated protection in NSC34 and NSC34 SOD1^G93A^ cells.** (**a**) PRDX3 protein levels of NSC34 cells transduced with LV-PRDX3 at 3 dpt relative to endogenous levels of untransduced cells (*N* = 3, mean ± SEM). (**b**) Representative western blot (25 μg of total protein per lane) of actin (47 kDa) and PRDX3 (27 kDa). (**c**) Time course of PRDX3 protein expression normalized to endogenous levels in NSC34 cells after transduction with LV-PRDX3 (MOI = 20). (**d**) NSC34 cell survival as determined by MTT assay at 5 dpt with LV-PRDX3 or LV-lacZ (MOI = 20) under basal conditions or after stress induction (two-way analysis of variance, Bonferroni's post-hoc test, *P* < 0.05, *N* = 5, mean ± SEM). (**e**) NSC34 cellular oxidative stress levels determined for the same conditions as in **d** relative to the oxidative stress levels of the untransduced cells through the DCF assay (one-way analysis of variance, Tukey's post-hoc test, *P* < 0.05, *N* = 5, mean ± SEM). (**f**) NSC34 SOD1^G93A^ cell survival and (**g**) NSC34 SOD1^G93A^ cellular oxidative stress levels, at 7 dpt with LV-PRDX3 or LV-lacZ (MOI = 40) under basal conditions (one-way analysis of variance, Tukey's post-hoc test, *P* < 0.05, *N* = 5, mean ± SEM). (**h**) Each cell survival or oxidative stress assay was undertaken after verification of PRDX3 protein overexpression by western blot as shown in representative blots of NSC34 and NSC34 SOD1^G93A^ cells. *P ≤ 0.05, **P ≤ 0.01, ***P ≤ 0.001. dpt, days post transduction; MOI, multiplicity of infection; PRDX3, peroxiredoxin 3.

*LV-PRDX3–mediated significant protection against oxidative stress.* After transduction of NSC34 cells with the lentiviral vector expressing PRDX3, quantification of transgene expression was assessed to determine optimal conditions defined as the maximum overexpression achieved at the lowest multiplicity of infection (MOI) without observing any cellular toxicity. NSC34 cells transduced at an MOI of 20 exhibited a threefold PRDX3 overexpression with this protein increase remaining stable for at least 2 weeks (**Figure 1a**–**c**). To explore the neuroprotective potential of PRDX3 overexpression, the transduced NSC34 cells were subjected to oxidative stress induction and the cell survival was determined by the MTT assay. After induction of oxidative stress–mediated cell death through serum deprivation, the increase in survival of cells transduced with LV-PRDX3 was 92% (*N* = 4, *P* < 0.001) compared with untransduced cells and 61% (*N* = 4, *P* < 0.01) compared with LV-lacZ (β-galactosidase gene)–transduced cells (**Figure 1d**). The effect of higher levels of oxidative stress in the cells was evaluated by the administration of menadione for 6 hours, which acts by generating reactive oxygen species in multiple subcellular compartments through a redox cycling mechanism.^23^ NSC34 cells transduced with LV-PRDX3 and treated with menadione exhibited increased cell survival, even though the difference compared with the untransduced cells did not reach statistical significance. This was probably due to the increased toxicity seen (76% cell death was seen in the untransduced cells), which was beyond the compensatory upregulation of PRDX3 as the NSC34 cells are particularly vulnerable to oxidative stress. More importantly, virally delivered *PRDX3* induced a 68% increase (*N* = 4, *P* < 0.001) in cell numbers under basal conditions compared with untransduced cells, and a 35% increase (*N* = 4, *P* < 0.05) compared with NSC34 cells transduced with LV-lacZ, with the growth rate of the PRDX3-transduced cells being greater than controls.

Oxidative stress levels were also measured by the dichlorofluorescein (DCF) assay to further investigate the effect of the virally delivered *PRDX3* gene (**Figure 1e**). In particular, LV-PRDX3 treatment led to a consistent and significant 30% decrease in oxidative stress compared with the untransduced cells under basal conditions (*N* = 5, *P* < 0.01), and after serum deprivation (*N* = 5, *P* < 0.001) reaching a 40% reduction (*N* = 5, *P* < 0.01) when cells were treated with 10 µmol/l of menadione. The oxidative stress levels between untransduced cells and cells transduced with the viral control LV-lacZ were comparable throughout.

Having evidence of the protective ability of virally delivered PRDX3 in NSC34 cells, the vector was then tested in the extensively characterized ALS tissue culture model, NSC34 cells stably expressing human SOD1^G93A^.^24^ In these cells, a threefold PRDX3 protein overexpression was accomplished at 7 days after transduction with an MOI of 40 (MOI = 20 afforded only 1.5-fold protein increase; data not shown). As the oxidative stress response mechanism of NSC34 SOD1^G93A^ cells is already compromised leading to decreased viability, there was no need to induce additional stress and the cells were studied only under basal conditions (**Figure 1f**–**h**). The cells overexpressing PRDX3 displayed a statistically significant 27% increase in cell survival compared with controls, and a significant 42% reduction in oxidative stress (*N* = 5, *P* < 0.001). These results demonstrated that virally delivered PRDX3 not only reduced the oxidative stress in non-transgenic cells upon stress and also cells carrying an ALS-causing *SOD1* mutation, but furthermore, it protected cells from oxidative stress–mediated cell death. Thus, the *PRDX3* gene was taken forward for additional studies in the *in vivo* model of ALS.

**Figure 2:**
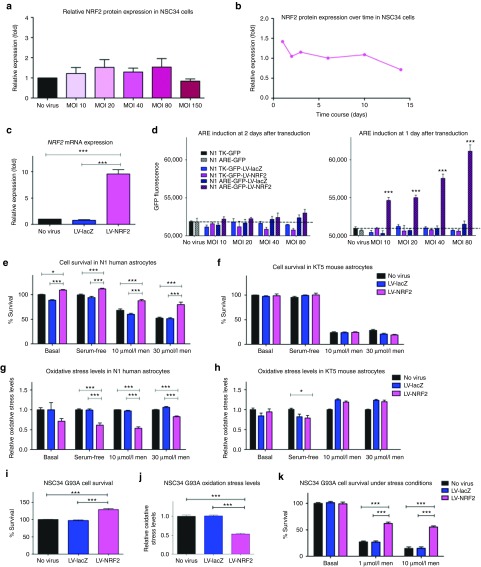
**LV-NRF2–mediated protection in neuronal and astrocytic cells.** (**a**) NRF2 protein levels of NSC34 cells transduced with LV-NRF2 at 3 dpt relative to endogenous levels of untransduced cells (*N* = 3, mean ± SEM). (**b**) Time course of NRF2 protein expression normalized to endogenous levels in NSC34 cells after transduction with LV-NRF2 (MOI = 40). (**c**) *NRF2* mRNA levels in NSC34 cells normalized to endogenous actin levels after transduction with LV-lacZ or LV-NRF2 (5 dpt, MOI = 40; one-way analysis of variance, Tukey's post-hoc test, *P* < 0.05, *N* = 3, mean ± SEM). (**d**) GFP fluorescence measured in human N1 astrocyte cell line carrying GFP under the TK promoter (solid bars) or under the ARE promoter (checkered bars) after transduction with LV-lacZ or LV-NRF2 at 1 and 2 dpt (two-way analysis of variance, Bonferroni's post-hoc test, *P* < 0.05, *N* = 3, mean ± SEM). (**e**) Human N1 astrocyte survival or (**f**) mouse KT5 astrocyte survival, as determined by MTT assay and normalized to untreated untransduced cells, at 3 dpt with LV-NRF2 or LV-lacZ (MOI = 40) under basal conditions or after stress induction (two-way analysis of variance, Bonferroni's post-hoc test, *P* < 0.05, *N* = 3, mean ± SEM). (**g**) Human N1 astrocyte or (**h**) mouse KT5 astrocyte, cellular oxidative stress levels determined for the same conditions as in **e**,**f** relative to the oxidative stress levels of the untransduced cells through the DCF assay (one-way analysis of variance, Tukey's post-hoc test, *P* < 0.05, *N* = 3, mean ± SEM). (**i**) Cell survival or (**j**) oxidative stress levels, of NSC34 SOD1^G93A^ after transduction of LV-lacZ or LV-NRF2 (7 dpt, MOI = 40) normalized to values of untransduced cells (one-way analysis of variance, Tukey's post-hoc test, *P* < 0.05, *N* = 5, mean ± SEM). (**k**) Cell survival of NSC34 SOD1^G93A^ under basal and stress conditions after transduction of LV-lacZ or LV-NRF2 (7 dpt, MOI = 40) normalized to values of untreated untransduced cells (two-way analysis of variance, Bonferroni's post-hoc test, *P* < 0.05, *N* = 3, mean ± SEM). *P ≤ 0.05, ***P ≤ 0.001. ARE, Antioxidant response element; dpt, days post transduction; GFP, green fluorescent protein; Men, menadione; MOI, multiplicity of infection; NRF2, nuclear factor erythroid 2–related factor 2; TK, thymidine kinase.

*LV-NRF2–mediated significant protection against oxidative stress. LV-NRF2 increases cell survival in both motor neuronal-like and astrocytic cell lines*: The evaluation of NRF2 as a potential therapeutic target for ALS started with characterization of the viral vector construct and NRF2 protein expression. Despite a significant increase in mRNA levels after transduction with LV-NRF2, a consistent increase in protein expression was not observed at the time points tested (**Figure 2a**–**c**). After further testing and optimization procedures (such as using various α-NRF2 antibodies with different epitopes, changing western blot conditions, isolating nuclear fractions for NRF2 detection, using the HEK293 cells for transduction/transfection and protein detection), it was considered likely for an inherent issue with NRF2 overexpression to occur possibly due to a strict control by the cell of this transcriptional regulator.

Thus, a different approach was taken in respect to the LV-NRF2 studies using reporter cell lines. After the dissociation of NRF2 from KEAP1 and its translocation to the nucleus, it binds to the ARE in the promoter region of detoxifying genes and antioxidant enzymes activating their expression.^16,17^ Induction of ARE-dependent transcription would signify that NRF2 activation was accomplished. In the absence of motor neuronal-like reporter cell lines, two astrocyte reporter cell lines were used; one stably expressing recombinant green fluorescent protein (GFP) under the control or the ARE promoter, and one under the control of thymidine kinase promoter as an additional control. The GFP fluorescence was measured over time in untransduced cells representing the background fluorescence, and also in LV-lacZ– or LV-NRF2–transduced cells (**Figure 2d**). At the 2nd day after transduction, a dramatic increase in fluorescence was seen in cells transduced with LV-NRF2 and expressing GFP under the ARE promoter, and this increase was seen for as long as the cells could be kept in culture (at least 5 days after transduction), with a positive correlation with the MOI used.

A possible way to limit the tight regulation of NRF2 might be the use of a cell line from a different species, as the small variations in the sequence of human and mouse *NRF2* might limit its susceptibility to regulation in a mouse or human cell line respectively. Thus, human and mouse astrocyte cells (astrocytes were used to the lack of a motor neuronal cell line of human origin) were transduced with the LV-NRF2 vector that contains the mouse *NRF2* gene, and cell survival and oxidative stress levels were determined under basal and stress conditions (**Figure 2e**–**h**). As expected, human cells more easily overexpressed the mouse NRF2 offering a protection against oxidative stress, whereas the mouse cells regulated NRF2 strictly and thus inhibited its overexpression. In human astrocytes, there was a small but significant increase of 10% under basal and serum deprivation conditions in LV-NRF2–transduced cells compared with controls with this increase reaching 30 and 50% in the harsher stress condition of 10 and 30 µmol/l menadione respectively, whereas no effect on the cell survival was seen on the mouse astrocytes (**Figure 2e**,**f**). Similarly, in human astrocytes transduced with LV-NRF2 a reduction (albeit not significant) in oxidative stress levels is seen under basal conditions, whereas after stress induction the oxidative stress levels are decreased by 40, 20, and 45% for conditions of serum withdrawal, 10 μmol/l and 30 μm menadione respectively (**Figure 2g**). In mouse astrocytes, despite the significant but small decrease in oxidative stress levels seen after serum withdrawal, no consistent reduction was seen between NRF2-transduced cells and controls (**Figure 2h**).

Despite the difficulties in detecting NRF2 protein overexpression, the effect of the LV-NRF2 in NSC34 SOD1^G93A^ cells was investigated, as an effect might be seen due to the *NRF2* downregulation^11^ and the impairment of proteasome^10^ in the presence of mutant SOD1. Interestingly, NSC34 SOD1^G93A^ cells transduced with LV-NRF2 (MOI = 40) showed a significant 29% increase in cell survival and a corresponding 54% decrease in oxidative stress compared with LV-lacZ–transduced or –untransduced cells (*N* = 5, *P* < 0.001) at 7 days after transduction (**Figure 2i**,**j**). In addition, it was considered whether LV-NRF2 could protect the already compromised NSC34 SOD1^G93A^ cells from additional oxidative stress and thus, NSC34 SOD1^G93A^ cells were further stressed by menadione and the relative survival rate was measured by MTT (**Figure 2k**). When the cells were stressed with 1 µmol/l menadione, the cell survival of the LV-NRF2–transduced cells increased by 130%, whereas when stressed with 10 µmol/l menadione the cell survival reached a 373% increase compared with controls. The greatly increased cell survival in the cells expressing virally delivered NRF2 highlights the therapeutic potential of this gene.

**Figure 3:**
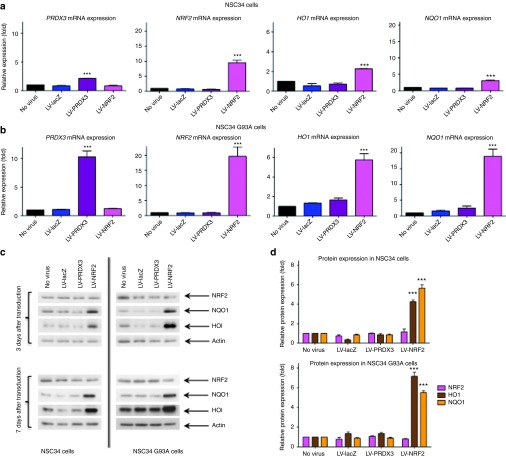
**Expression of downstream genes of the NRF2 pathway after transduction with LV-NRF2 vector.** mRNA levels of *PRDX3*, *NRF2*, *HO1* and *NQO1* normalized to endogenous actin levels as assessed by quantitative PCR in (**a**) NSC34 and (**b**) NSC34 SOD1^G93A^ cells transduced with LV-lacZ, LV-PRDX3 or LV-NRF2 (7 dpt, MOI = 40; one-way analysis of variance, Tukey's post-hoc test, *P* < 0.05, *N* = 3, mean ± SEM). (**c**) Representative western blots of NRF2 (68 kDa), NQO1 (31 kDa) and HO1 (35 kDa) protein expression levels in NSC34 and NSC34 SOD1^G93A^ cells after transduction with LV-lacZ, LV-PRDX3 or LV-NRF2 (3 and 7 dpt, MOI = 40, *N* = 3). (**d**) Quantification through densitometric analysis of protein expression of NRF2, NQO1 and HO1 expressed to the endogenous levels of untransduced cells and normalized to actin levels after LV-lacZ, LV-PRDX3 or LV-NRF2 transduction (7 dpt, MOI = 40; two-way analysis of variance, Bonferroni's post-hoc test, *P* < 0.05, *N* = 3, mean ± SEM). ***P ≤ 0.001. dpt, days post transduction; HO1, hemeoxygenase 1; MOI, multiplicity of infection; NQO1, NAD(P)H quinine oxidoreductase 1; NRF2, nuclear factor erythroid 2–related factor 2; PRDX3, peroxiredoxin 3.

*Downstream gene expression changes after NRF2 modulation*: To further elucidate the mechanism of action of NRF2, the protein and mRNA levels of several downstream genes of the NRF2 pathway were studied in both transgenic and non-transgenic cell lines (**Figure 3a**–**d**). Two well-known antioxidants under the control of NRF2 were selected, *HO1* and *NQO1*, and PRDX3 was also included as an added control of an antioxidant that does not act through the NRF2 pathway. It is particularly interesting to note that although in both NSC34 and NSC34 SOD1^G93A^ cell lines there is an increase in *NRF2* mRNA, in both cell lines there is no detectable protein overexpression, total or nuclear. Furthermore, the level of mRNA increase in NSC34 and NSC34 SOD1^G93A^ is very different not only in *NRF2* (10-fold and 20-fold respectively), but also in PRDX3 (2-fold and 10-fold respectively), HO1 (2-fold and 6-fold respectively, and NQO1 (3-fold and 19-fold respectively) (**Figure 3a,b**). These differences between the two cell lines might demonstrate a differential affinity for the viral transduction as all endogenous levels are comparable, leading to greater levels of the virally delivered target gene and subsequently greater activation of downstream genes.

It is noteworthy that this mRNA increase of the downstream genes corresponds to a protein overexpression in both cell lines. The protein levels in NSC34 and NSC34 SOD1^G93A^ cells after LV-NRF2 transduction at 7 days after transduction were shown to be a fourfold and sevenfold increase respectively for HO1, a 5.5-fold increase for NQO1 for both cell lines (**Figure 3c**,**d**). This activation of at least two downstream genes renders the viral delivery of NRF2 a very attractive strategy for treating ALS, as with the delivery of one gene, multiple other antioxidant and cytoprotective enzymes could be induced. Thus, evaluation of the effect of NRF2 overexpression in the *in vivo* model of ALS was considered worthwhile.

### AAV6-mediated PRDX3 and NRF2 modulation *in vivo*

**Figure 4:**
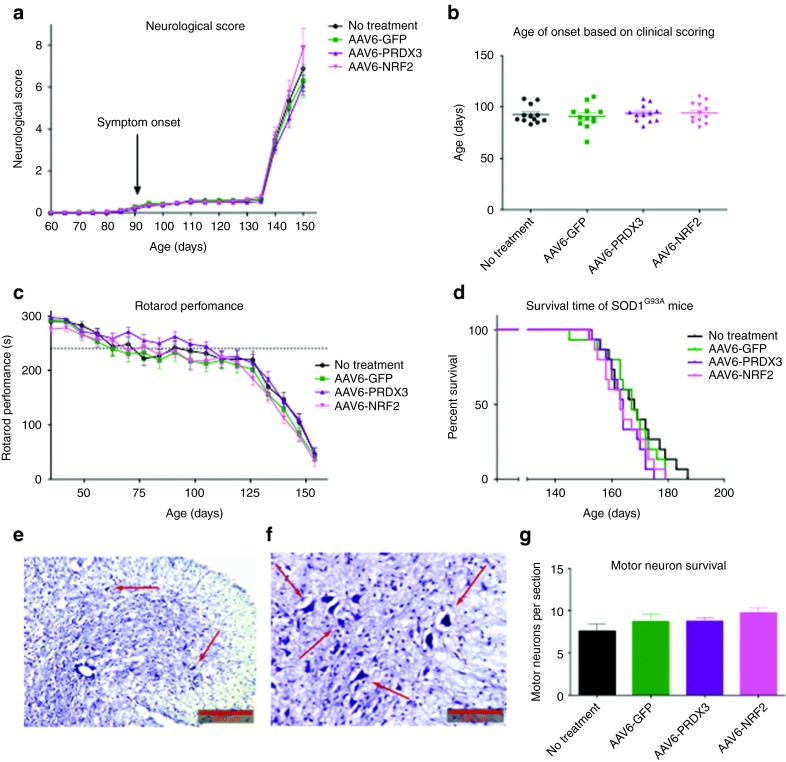
**Efficacy study of AAV6-PRDX3 and AAV6-NRF2 in SOD1^G93A^ mice.** (**a**) Changes in neurological score (average score per group) over time in experimental mice (*N* = 15 mice per group, two-way analysis of variance, Bonferroni's post-hoc test, *P* < 0.05, mean ± SEM). (**b**) Comparison of the average age of onset between experimental groups as calculated by neurological scoring (*N* = 15 mice per group, two-way analysis of variance, Bonferroni's post-hoc test, *P* < 0.05, mean ± SEM). (**c**) Rotarod performance (*i.e.,* ability of mice to run in seconds) over time between experimental groups with the dashed line depicting a 20% reduction in rotarod performance (*N* = 15 mice per group; two-way analysis of variance, Bonferroni's post-hoc test, *P* < 0.05, mean ± SEM). (**d**) Kaplan–Meier plot to compare lifespan between all experimental groups (*N* = 15 per group; *P* < 0.05). (**e**,**f**) Representative lumbar spinal cord sections from untreated control SOD1^G93A^ mice, fixed and stained with Nissl substance. Scale bars are shown at the bottom right corner (**e**, 200 μm; **f**, 100 μm). (**g**) Quantification of motor neurons (average number per section per group) by counting across all groups (*N* = 5; one-way analysis of variance, Bonferroni's post-hoc test, *P* < 0.05, mean ± SEM). AAV6, adenoassociated virus serotype 6; GFP, green fluorescent protein; NRF2, nuclear factor erythroid 2–related factor 2; PRDX3, peroxiredoxin 3.

To evaluate the viral delivery of *PRDX3* or *NRF2* as a therapeutic strategy of ALS, an *in vivo* study was undertaken in the SOD1^G93A^ mouse model. The two genes were transferred to an AAV6 viral construct to accomplish spinal cord motor neuronal delivery through intramuscular injections as previously undertaken,^25,26^ and as a less invasive procedure than intraspinal, intrathecal, or intraventricular injections. Although the target tissue was the motor neuronal population, concomitant muscle transduction might confer some protective effects on the neuromuscular junction. An AAV6-GFP vector was used as a viral control, and an untreated group was included to monitor the disease progression without any intervention. Unfortunately, no differences were found between the experimental groups and control groups for any outcome measurement including neurological score, age of onset, rotarod performance, survival or motor neuron cell survival^27^ (**Figure 4a**–**g**). Similarly, no differences were found for weight distribution, disease survival or various gait measurements as analyzed by the CatWalk system (data not shown). To elucidate whether this lack of effect was due to inefficient delivery or due to ineffectiveness of the therapeutic agents, a quantification of the transduction efficiency was necessary.

**Figure 5:**
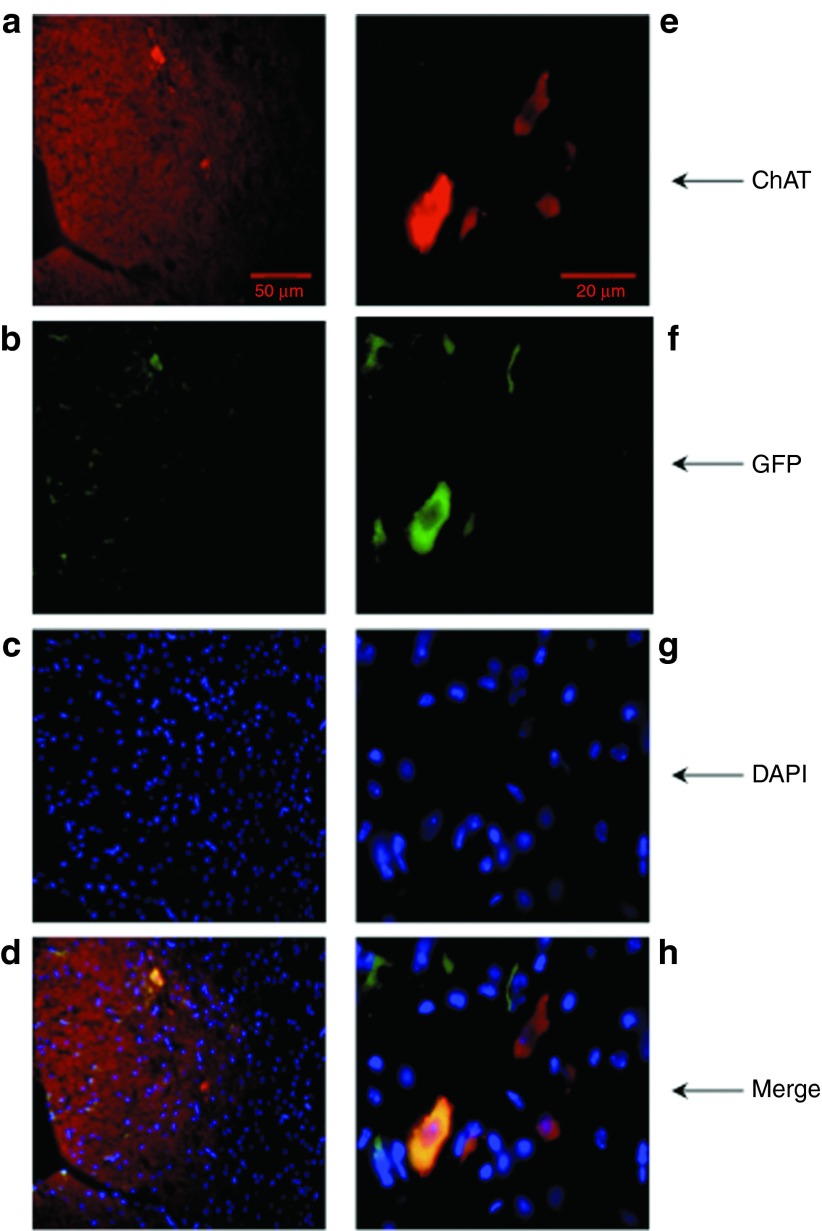
**Transduction efficiency of spinal motor neurons in SOD1^G93A^ mice.** Lumbar spinal cord sections from SOD1^G93A^ mice treated with AAV6-GFP were fixed and labeled with (**a**,**e**) ChAT antibody as a neuronal marker, (**b**,**f**) GFP antibody as an indicator of transduction, and (**c**,**g**) DAPI for nuclear visualization. Pictures on the left (**a**–**d**) were taken using ×20 magnification (50 μm scale bar), and on the right (**e**–**h**) using ×63 magnification (20 μm scale bar). AAV6, adenoassociated virus serotype 6; ChAT, choline acetyltransferase; GFP, green fluorescent protein.

A previous pilot study undertaken by our lab had shown that, although western blot analysis verified very high protein overexpression in muscle, the dynamic range of the technique did not allow the quantification of protein expression in spinal cord (**Supplementary Figure** S1). As such, a histological analysis was performed of the lumbar spinal cord. Lumbar spinal cord sections from three mice, which were sacrificed after reaching the animal study endpoint, were stained with fluorescent antibodies against GFP (green) and choline acetyltransferase (red) as a neuronal marker, and analyzed by fluorescence microscopy to detect the AAV6-GFP–transduced motor neurons (**Figure 5**). The transduction efficiency was calculated as the ratio of transduced motor neurons to total motor neurons, and as seen in **Table 1**, <5% of the surviving lumbar motor neurons were transduced. This low level of gene transfer could adequately explain the absence of any therapeutic effect in the *in vivo* model.

## Discussion

The therapeutic potential of *PRDX3* and *NRF2* genes was demonstrated in a tissue culture model of SOD1-related ALS suggesting that they could be used as therapeutic interventions. In agreement with other reports, we found that viral delivery of PRDX3 protected the cells against oxidative stress insults supporting its application to *in vivo* systems.^28^ To further support the therapeutic potential of virally delivered PRDX3 to motor neurons, the distribution of PRDX3 in neural cell types should be taken into account. A study investigating the differential expression patterns of all the members of the peroxiredoxin family reported histological evidence that no PRDX3 immunoreactivity was seen in astrocytes or glial cells in the brain.^29^ This supports our hypothesis that direct delivery to the motor neurons might offer protection against oxidative stress. Furthermore, decreased PRDX3 levels have been observed in brain regions from patients suffering from Down syndrome, Parkinson's and Alzheimer's disease, suggesting both a common role for oxidative stress in different neurological disorders and a potential common approach to treatment.^30,31^

The importance of NRF2 as a therapeutic strategy is highlighted by the fact that in primary motor cortex and spinal cord post-mortem tissue samples from patients with ALS a reduction of NRF2 at both protein and mRNA levels, and a concomitant increase in *KEAP1* mRNA levels was shown.^21^ Importantly, specific NRF2 overexpression in astrocytes of ALS mice resulted in decreased glial reactivity, delayed disease onset and increased mouse survival.^22^ Furthermore, a neuronal cell line expressing mutant TDP-43 exhibited increased neurite outgrowth when treated with MG132 by inducing an upregulation in *HO1*.^32^ Although the increased expression of HO1 was independent of the NRF2 pathway, HO1 is regulated by NRF2 and an induction of *NRF2* could in turn lead to HO1 overexpression. As in this project the viral delivery of NRF2 lead to overexpression of downstream genes of the NRF2 pathway, neuroprotection could potentially be induced independently by other antioxidant genes of the pathway.

Nonetheless, it has proven very difficult to consistently overexpress the NRF2 protein, and the results presented in this paper suggest that mutant SOD1-induced alteration of the gene expression profile of neuronal cells allows sufficient upregulation of NRF2 to confer neuronal protection. Nonetheless, without a clear understanding of the mechanism of NRF2 regulation, it is difficult to evaluate the clinical application of such an approach. Although the mechanism of NRF2 regulation is not clear yet despite the extensive research, it has been suggested that a very rapid response to stress changes the balance of the nuclear import and export of the protein promoting nuclear accumulation and thus subsequent transcriptional activation.^33^ In particular, upon cellular treatment with an NRF2 inducer, NRF2 accumulated in the nucleus within 15 minutes, and between 1 and 4 hours after treatment NRF2 started exiting the nucleus reaching normal cytosolic localization in a total of 8 hours.^33^ Such a dynamic regulation of NRF2 could impose difficulties in detecting it when considering its rapid turnover.

Thus, in the case of the non-transgenic NSC34 cells, it is possible that after transduction with LV-NRF2, an autoregulatory loop was initiated shortly after the expression of the viral NRF2, and it has been observed that transgene expression can be detected as early as 2 days after transduction. Protein levels were not evaluated after the oxidative stress insult, and the stress induction might have been too long for quantification of overexpression (at least 1 hour). On the other hand, the inconsistent NRF2 protein levels in NSC34 SOD1^G93A^ cells might be explained by a continuous cycle of NRF2 induction and inhibition. In this case, the increased oxidative stress caused by mutant SOD1 could upregulate NRF2, and its upregulation in combination with the virally delivered NRF2 could induce its inhibition. This phenomenon could provide short “bursts” of NRF2 activity, and induction of ARE transcription. As the downstream genes activated by NRF2 do not have such a readily activated degradation pathway, the cytoprotection seen could be due to their transcription, but further investigation is needed to substantiate this.

Although the work presented here cannot describe conclusively a mechanism of action for NRF2, it provides pointers for further investigation. A tight time course experiment, in which protein lysates are extracted from cells transduced with LV-NRF2 every few hours, will provide a clear picture of the protein expression over time, and also how quickly it is suppressed by the cell. Furthermore, concomitant inhibition of genes negatively regulating NRF2 (such as *KEAP1*) will give evidence for the design of strategies for inducing a stable protein overexpression. This could be accomplished either by the co-transduction of cells using two viral vectors (for example, one expressing NRF2 and one expressing siRNA against *KEAP1*), or by a bidirectional vector. Alternatively, known inducers of NRF2 could be used in combination with the viral vectors to examine their effect on protein regulation

When the *PRDX3* and *NRF2* genes were taken forward to the animal model, no beneficial effect was seen compared with controls (untreated or AAV6-GFP–treated mice). However, <5% of the spinal motor neurons were transduced and such a low transduction efficiency would be unlikely to be sufficient to induce a therapeutic effect. Moreover, it is likely that the actual percentage of transduced neurons is even lower assuming the transduced neurons survive longer, as by the end-stage of the disease there is a substantial motor neuron loss.^34^ When assessing the transduction efficiency in the muscle, it was seen that all muscle groups injected expressed the transgene very strongly (**Supplementary Figure S1**). Although the transgene expression was not widespread within the whole muscle and it persisted in an area surrounding the injection site, the expression was so strong that the muscle injected with AAV6-GFP could be recognized by naked eye. This observation indicated that overexpression of NRF2 and PRDX3 in the muscle is not likely to affect the disease course in SOD1^G93A^ transgenic mice.

Recombinant AAV are capable of neuronal retrograde transport when delivered to skeletal muscle,^35^ and AAV6 has been shown to induce a widespread skeletal muscle transduction.^36^ Thus, the motor neuronal population could be targeted by administering the viral vector directly into muscle. However, there is increasing evidence that the toxicity caused by mutant SOD1 is not cell-autonomous, and a therapeutic strategy targeting motor neurons might not be sufficient to influence the disease course.^37,38,39,40,41^ When intramuscular injections of AAV6 expressing shRNA for silencing mutant SOD1 in the hind limbs were performed, 3.2% transduction efficiency was accomplished in the motor neurons of the lumbar spinal cord.^42^ As expected, the disease progression was not altered, even though the protein levels in the muscle were reduced by >50%. The authors then tried to administer body-wide intramuscular injections in neonatal SOD1^G93A^ mice in an attempt to increase the transduction efficiency.^26^ Although this approach resulted in enhanced delivery, motor neuronal protection, and also muscle atrophy attenuation, it did not confer a therapeutic impact on disease progression.

Hence, it is likely that a motor neuronal delivery might not be sufficient to modify the disease, and a more widespread CNS administration may be needed. With the development of the AAV9 serotype that appears to cross the blood–brain barrier, a new delivery option has been presented with unique advantages. It would be also of great interest to identify whether a systemic or CNS-targeted delivery could reach therapeutic levels. Parallel experiments in mice comparing a CNS delivery by administering the viral vector through intraspinal or intrathecal injection, and systemic delivery by administration though intravenous injections, will elucidate the most appropriate protocol, and the importance of a widespread therapeutic strategy.

## Materials and Methods

***Viral vector production.*** The cDNAs encoding for the reporter gene *lacZ*, the *GFP* gene and the mouse genes of interest (*PRDX3* and *NRF2*) containing a Kozak consensus sequence (GCCACC) were subcloned either into the pIRES or TOPO pCRII vectors. They were then subcloned using appropriate sites in the destination expression viral vectors (SIN-PGK-cPPT-WHV vector for the LV viral vectors or ScAAV2-CMV-GFP for the AAV6 viral vectors). All constructs were confirmed by restriction enzyme digestions and sequencing. LV pseudotyped with the vesicular stomatitis virus-G envelope protein were generated by calcium phosphate 4-plasmid (SIN-W-PGK, pCMVΔR8.92, pMD.G, and pRSV-Rev) cotransfection of HEK293T cells as previously described.^43^ The titers of the viral stocks were obtained by p24 (capsid antigen) ELISA assay (ZeptoMetrix, Buffalo, NY) following manufacturer's recommendations with serial dilutions of LV vectors. The AAV6 expressing PRDX3 was generated in house by calcium phosphate 2-plasmid (AAV6-PRDX3 and pDGM6) cotransfection of HEK293T cells as previously described.^36^ Vector purification was accomplished by affinity chromatography (AKTAprime plus purification system; GE Healthcare, Amersham, UK) using a heparin column (HiTrap heparin HP Column; GE Healthcare) and the crude viral vector solution was collected after elution in HBSS containing 400 mmol/l NaCl.^44^ After filtering through 0.2 µm filter and concentrating the viral solution by ultra-centrifugation through a sucrose cushion (Sigma, Dorset, UK), the pellets were resuspended in 230 µl HBSS. An aliquot of 30 µl was kept for Coomassie staining and analysis of titre through quantitative PCR. All preparations with a titre <1 × 10^12^ viral particles per ml were discarded, whereas the rest were diluted and aliquoted to 120 μl with a titre of 1 × 10^12^ viral particles per ml. AAV6-NRF2 and AAV6-GFP viral vectors were prepared by Virapur (Virapur, San Diego, CA) and by the Gene Therapy Centre (University of North Carolina, Chapel Hill, NC), and the titres of both viral vectors were verified by quantitative PCR, diluted if needed and aliquoted to 120 μl (1 × 10^12^ viral particles per ml titre).

***Cell lines and lentiviral transduction.*** NSC34 cells, a hybrid mouse motor neuron-neuroblastoma cell line^45^ were used for the cell assays and western blots. NSC34 cells stably transfected with the G93A mutant human SOD1 cDNA were also used and cultured under the selection pressure of G418 antibiotic.^24^ HEK293T cells were used for LV production, and HEK293D cells (kindly provided by Jeffrey Chamberlain, University of Washington School of Medicine, Seattle, WA) were used for the AAV production. Human N1 astrocytes (1321N1 cell line; ECACC, Salisbury, UK) and mouse KT5 astrocytes (JCRB Cell Bank, Ibaraki City, Osaka, Japan) were also used for the oxidative stress levels measurement assay. Furthermore, human N1 astrocyte reporter cell lines were used, which were stably transfected with the GFP either under the thymidine kinase promoter or under the ARE promoter. Lentiviral transduction efficiency of all cell lines was assessed by immunofluorescence and cell counting, and standardized per cell line. Appropriate viral vector concentrations were used to achieve the maximum non-toxic transduction ratio of 85–90% for NSC34 and NSC34 SOD1^G93A^ cells, and 95–100% for N1 and KT5 astrocytes.

***Oxidative stress levels measurement and cell viability assays.*** Cellular oxidative stress was induced by serum withdrawal or menadione for 6 hours. Reduction of 0.1 volumes of 5 mg/ml 3-(4,5-dimethylthiazol-2-yl)-2,5-diphenyl tetrasodium bromide (MTT; Sigma) by cellular dehydrogenases was used to quantify the number of living cells in 96-well plates. After 1 hour incubation at 37°C, 1 volume of DMF/SDS (20% SDS in 50% DMF, pH 4.7) was added to each well at room temperature on a shaking platform for 1 hour and the absorbance was determined at 595 nm. For measurement of the oxidative stress levels, the DCF (10 mmol/l 6-carboxy-2′,7′-dichloro-dihydrofluorescein diacetate di (acetoxymethyl) ester, Invitrogen, Paisley, UK) dye was added to cells at a final concentration of 5 µmol/l. Oxidative stress levels were measured by the increase in fluorescence of the DCF dye (excitation at 485 nm and emission at 530 nm), and a read was taken before the addition of the dye to account for background fluorescence. The toxicity of the assay was monitored by adding ethidium homodimer-1 dye (Invitrogen). It was added concurrently with the addition of the DCF dye at a final concentration of 0.33 µmol/l, and the fluorescence was measured at excitation wavelength of 530 nm and emission wavelength of 645 nm. To account for the experimental variation in plating or differential growth rate of cells, the data were normalized according to the cell number obtained by the MTT assay performed on the same plates after the completion of the DCF assay.

***Quantitative PCR.*** Total RNA was extracted with the SV total RNA isolation system (Promega, Southampton, UK), and cDNA was synthesized using SuperScript III First-Strand Synthesis System (Invitrogen) according to manufacturers' protocols. Quantitative PCR reactions were performed with SybrGreen Master Mix (Agilent Technologies, Cheshire, UK) and run on an MX3000P Real-Time PCR System (Stratagene, Cheshire, UK). To detect the expression of transgenes, determine AAV6 titres and detect the expression of downstream genes, the following primers were used: forward NRF2 (150 nmol/l), 5′-TGGAGGCAGCCATGACTGA-3′ reverse NRF2 (300 nmol/l), 5′-CTGCTTGTTTTCGGTATTAAGACACT-3′ forward PRDX3 (300 nmol/l), 5′-GCAGCTGCGGGAAGGTT-3′ reverse PRDX3 (150 nmol/l), 5′-GGCAGAAATACTCCGGGAAAT-3′ forward HO1 (900 nmol/l), 5′-CACTTCGTCAGAGGCCTGCTA-3′ reverse HO1 (900 nmol/l), 5′-GCGGTGTCTGGGATGAGCTA-3′ forward NQO1 (600 nmol/l), 5′-CGCCTGAGCCCAGATATTGT-3′ reverse NQO1 (300 nmol/l), 5′-ACTGCAATGGGAACTGAAATATCA-3′. β-actin was used as the housekeeping gene with the following primers: forward actin (900 nmol/l), 5′-ATGCTCCCCGGGCTGTAT-3′ reverse actin (300 nmol/l), 5′-CATAGGAGTCCTTCTGACCCATTC-3′.

***Western blotting.*** Proteins were extracted from cells or tissues, separated on 8.5–15% SDS-polyacrylamide gels depending on the molecular weight of the protein of interest, and transferred to a polyvinylidenedifluoride membrane (Merck Millipore, Billerica, MA). The following primary antibodies were used: mouse antibody to PRDX3 (Ab16751, Abcam, Cambridge, UK), goat antibody to NRF2 (Ab31163, Abcam), rabbit antibody to NQO1 (Ab80588, Abcam), rabbit antibody to HO1 (Ab13243, Abcam), and rabbit antibody to β-actin (Ab8227, Abcam). They were followed by horseradish peroxidase-conjugated secondary antibodies chosen according to the protein(s) of interest: rabbit antibody to goat (P0449, Dako, Ely, UK), goat antibody to mouse (170–6516, Biorad, Hemel Hempstead, UK), goat antibody to rabbit (P0448, Dako), and swine antibody to rabbit (P0217, Dako).

***Animals.*** All procedures were carried out according to the UK Home Office regulations. All mice used in this project were housed singly, were provided with standard chow and water ad libitum, and were exposed to an automated 12 hour light–dark cycle at 24 °C. The congenic C57BL/6J Tg (SOD1 G93A)1Gur (G93A-SOD1) mice (Jackson Laboratory, Bar Harbor, ME) were used,^46^ which were backcrossed onto the wild-type C57BL/6 mice (Charles River Laboratories, Wilmington, MA) for >10 generations. A priori power analysis showed that 15 animals per group was a sufficient sample size to detect a medium effect with an 80% power. Transgenic mice (genotyped according to the Jackson Laboratory protocols) were recruited in subgroups of four (one mouse per group; (i) no treatment, (ii) AAV6-GFP, (iii) AAV6-PRDX3, and (iv) AAV6-NRF2), and they were age-matched and if possible, litter-matched. A total volume of 120 μl of viral vector solution was injected intramuscularly per mouse (30 days of age ± 1 day) in various muscle groups (facial muscles, 20 μl; tongue, 10 μl; intercostal muscles, 15 μl; diaphragm, 15 μl; hind limbs, 60 μl) using isoflurane anesthesia. Multiple injection sites were used per muscle group with a maximum of 5 μl solution per site administered slowly using a 10 μl 33-Gauge Hamilton syringe (ESS Laboratory, Cranston, RI). Behavioral assessment and all *in vivo* tests were carried out blinded to treatment. All mice were weighed and scored twice per week until they reached 140 days of age, and from that point onwards they were weighed and scored daily. Rotarod tests were performed twice per week (2 trials per test, only best score was recorded). The rotarod was set to accelerate from 4 to 40 rpm (rotations per minute) in 300 seconds, and the latency to fall was recorded in seconds. The maximum time showing no motor impairment was defined as 300 seconds. In addition, gait analysis was performed using the automated footprint recording Catwalk system 7.1 (Noldus Information Technology, Wageningen, The Netherlands) every 10 days. Briefly, each mouse was allowed to run from one side to the other of a glass plate and a video camera underneath the glass plate recorded the video image of the run.

***Histological analysis.*** When transgenic mice reached the endpoint (*i.e.,* loss of righting reflex for >10 seconds or 30% weight loss from peak weight for 3 days), they were terminally anesthetized with pentobarbital and then transcardially perfused with phosphate-buffered saline supplemented with 5 units/ml heparin (Sigma) followed by 4% paraformaldehyde inphosphate-buffered saline. After overnight incubation in 4% paraformaldehyde, all tissues were cryoprotected in 30% sucrose in phosphate-buffered saline for 1 day and subsequently cryoembedded in optimum cutting temperature medium (Dako). Lumbar spinal cords were serially sectioned at 10 µm thickness, and five sections were mounted per slide. Every other slide was used to determine motor neuron counts under a light microscope using the Nissl stain (0.1% Cresyl Fast Violet; BDH, Lutterworth, UK) for a total of 20 mice (five per group). Immunohistochemistry was performed to detect transduced motor neurons using the lumbar spinal cords of three mice treated were AAV6-GFP, and incubating them concomitantly with 1:500 dilution of a rabbit antibody to GFP (AB290, Abcam) and 1:100 dilution of a goat antibody to choline acetyltransferase (AB144P; Merck Millipore). The secondary antibodies incubations were performed sequentially firstly with a donkey Alexa Fluor 568 antigoat antibody (A-11057, Invitrogen), and then with a goat FITC antirabbit antibody (111-095-003, Jackson ImmunoResearch, Suffolk, UK). The percentage of transduced neurons was determined by counting the cells exhibiting double staining. For western blot analysis, tissues were collected fresh after intracardiac perfusion with phosphate-buffered saline and snap frozen in liquid nitrogen.

***Statistical analysis.*** Power analysis was conducted using GPower 3.0 software (Dusseldorf University, Dusseldorf, Germany) and statistical analysis was performed using GraphPad Prism v5 (GraphPad, San Diego, CA). Statistical significance was determined by one- or two-way analysis of variance depending on the number of variables under investigation and on individual experiment, as stated in figure legends. In figures, the asterisk is used to denote the statistical significance (*P ≤ 0.05, **P ≤ 0.01, ***P ≤ 0.001), whereas its absence shows no statistical significance. In general, in cell survival assays the effect of the viral construct and the oxidative stress conditions were investigated concurrently to ensure that the number of untreated cells in the increased stress conditions is within the therapeutic window and the compensatory ability of each therapeutic intervention in question for subsequent comparisons. In DCF assays, the experimental design of the procedure allowed the monitoring of the toxicity, and thus only the effect of the viral construct per stress condition was analyzed.

**SUPPLEMENTARY MATERIAL** **Figure S1.** Gene transfer detection after viral delivery in post-mortem SOD1^G93A^ mouse tissues.

## Figures and Tables

**Table 1:**
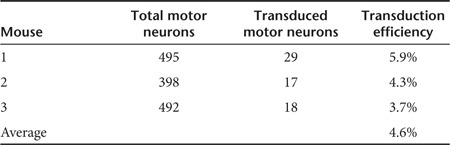
Transduction efficiency of lumbar motor neurons of SOD1^G93A^ mice
