# Supplementary figures and images for: Viral Delivery of Antioxidant Genes as a Therapeutic Strategy in Experimental Models of Amyotrophic Lateral Sclerosis
Source: Mol Ther. 2013 Jun 4;21(8):1486–96. doi: 10.1038/mt.2013.115 (PMC3734656; doi:10.1038/mt.2013.115)

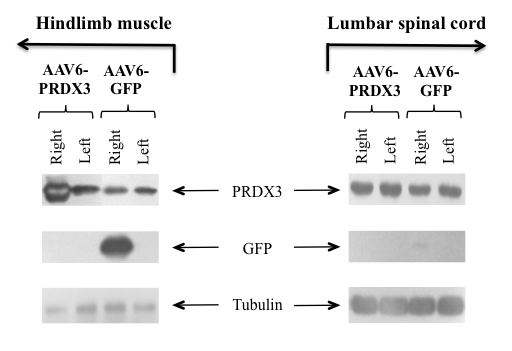

Supplement: Supplementary Information [file mt2013115x1.tiff]
